# Assessing the risk factors and management outcomes of ectopic pregnancy: A retrospective case-control study

**DOI:** 10.18502/ijrm.v21i5.13475

**Published:** 2023-05-12

**Authors:** Azadeh Tarafdari, Mahin Bandarian, Sedigheh Hantoushzadeh, Alireza Hadizadeh, Saeedeh Shahsavari, Maryam alsadat Razavi

**Affiliations:** ^1^Department of Obstetrics and Gynecology, Imam Khomeini Hospital Complex, Tehran University of Medical Sciences, Tehran, Iran.; ^2^Department of Obstetrics and Gynecology, Ziaeian Hospital, Tehran University of Medical Sciences, Tehran, Iran.; ^3^Research Center for Advanced Technologies in Cardiovascular Medicine, Cardiovascular Diseases Research Center Institute, Tehran University of Medical Sciences, Tehran, Iran.

**Keywords:** Contraception, Ectopic pregnancy, Intrauterine device, Levonorgestrel.

## Abstract

**Background:**

Ectopic pregnancy (EP) is the implantation of a fertilized egg outside the uterine cavity or in an unusual location. According to the clinical case reports, hormonal contraceptive failures may be related to emergency contraceptives and EP. EP may be treated medically, surgically, or expectantly. Currently, there is no consensus regarding whether a multiple- or double-dose regimen with methotrexate (MTX) or an additional dose could be more effective than a single-dose regimen.

**Objective:**

This study aimed to assess risk factors and treatment outcomes for EP.

**Materials and Methods:**

This case-control study was conducted in Tehran, Iran from March 2020 to March 2021. The case group was comprised of all EP-diagnosed cases (n = 191). Based on the levels of β-human chorionic gonadotropin, MTX was administered to stable individuals with no surgical indications. Risk factors were assessed through 2 control groups: intrauterine pregnancy (n = 190) and nonpregnant groups (n = 180).

**Results:**

The medical treatment significantly improved with an extra dose of MTX, especially in individuals with higher β-human chorionic gonadotropin concentrations and gestational age > 7.5 wk (p = 0.002). Considering risk factors, it is assumed that hormonal contraceptive failures, including both oral and emergency contraceptives, may increase the EP likelihood (p < 0.001).

**Conclusion:**

Based on our findings, we recommended an additional dose of MTX for subjects who are further along in their pregnancy. It is also concluded that failure of contraceptive pills increases the chances of EP.

## 1. Introduction

Ectopic pregnancy (EP) is the implantation of a fertilized egg outside the uterine cavity or in the scar resulting from a prior cesarean section. This complicated pregnancy has the potential to increase maternal morbidity and mortality. Even though the incidence of EP increased 6-fold between the 1970s and 1990s, recent research indicates that the statistics have remained relatively stable. EP still accounts for approximately 2% of all pregnancies and 10% of all pregnancy-related deaths (1-5). EP is diagnosed by a combination of symptoms, clinical examination, serial beta-human chorionic gonadotropin (β-hCG) measurements, and ultrasonographic imaging. Symptoms include abdominal pain and vaginal bleeding between the 6 ${}^{\text{th}}$ and 10 ${}^{\text{th}}$ wk of pregnancy (6).

Several factors have been linked to EP, with previous EP, pelvic surgery, pelvic inflammatory disease, Chlamydia trachomatis infection, and smoking is the most studied and well-known (7, 8). In recent years, however, it has been suggested that certain medications, specifically emergency contraceptive pills, are a major cause (9, 10). In addition, it has been hypothesized that ovulation induction and assisted reproductive technologies (ART) may also contribute to the development of EP (11-13).

The most common sites for the implantation of an ectopic gestational sac are the fallopian tubes, the cervix, the ovary, and the caesarian scar (14, 15). These pathologies necessitate a specific treatment; consequently, depending on the examinations, laboratory tests, and imaging, EP can be managed by observation, surgery, or medication (16-18). In cases of rupture, surgical removal of conception products is considered the definitive treatment for EP and is the method of choice. Surgical treatment includes salpingectomy, salpingostomy for tubal EP, and cesarean scar pregnancy curettage, among others (19-21).

Conservative therapies are recommended if the case meets the criteria for medical management, and the fallopian tubes are saved (1, 18, 22, 23). Medical methotrexate (MTX) regimens have a success rate of up to 93% for multidose protocols and 88% for single-dose therapy in tubal EP (1, 24). However, because single-dose MTX may fail, particularly at higher β-hCG concentrations ( > 5000 IU/l), a double-dose regimen may lower the risk while making the case less susceptible to adverse effects. Because both treatment protocols are prone to failure, an adjuvant dose may help reduce failure rates (23, 25, 26).

Considering all management approaches and possible risk factors, this study was designed to evaluate risk factors and treatment outcomes in women with EP.

## 2. Materials and Methods

In this retrospective case-control study 191 women who were diagnosed with EP and were referred to the obstetrics and gynecology clinic or emergency department of Imam Khomeini hospital complex, Tehran, Iran between March 2020 and March 2021 were enrolled. For control groups 190 pregnant women along with 180 non-pregnant women were enrolled. EP diagnosis was made using physical examination, serial β-hCG, and ultrasonographic imaging. Pregnant control group included patients with intrauterine pregnancy and without any anatomic or uterine abnormalities. All patients with concurrent diseases such as cancers and autoimmune diseases were excluded from this study.

Data were collected using the medical records at the hospital. In addition to being interviewed, subjects were asked to complete a structured questionnaire assessing their past medical and surgical history, infertility and ART, and use of contraceptive pills and devices. The nonpregnant control subjects were women with regular sexual activity and no nonmedical causes of infertility. The choice of treatment according to the protocol was based on β-hCG concentration and if the patients were either hemodynamically unstable or showed signs of rupture or had any indications such as failure to medical treatment they were candidate for surgery of the patients (Table I).

Moreover, pregnant control subjects were selected from those without abnormalities or anatomical malformations that could confound the analysis.

This study was designed with 2 control groups, intrauterine and nonpregnant women, to assess the relationship between EP and risk factors, particularly contraceptives used during the most recent ovulation cycle. Both control groups consisted of sexually active women of reproductive age. The cases and controls were matched regarding age ( ± 5 yr), gravidity, and parity in a ratio of 1:1.

**Table 1:** Medical treatment protocol for EP. Any case that faces rupture or has any kind of surgical management indication is considered a failure of medical treatment


**Protocol **	**Evaluation**
**Single dose** **(MTX 50 mg/m^2^ IM)** **(β-hCG < 4999 IU/L)**	A simgle dose of MTX on day 1 measurement of β-hCG on days 1, 4, and 7 then β-hCG is measured weekly until undetectable and if the difference is below 15%, an extra dose is given then β-hCG is measured weekly until undetectable
**Double dose** **(MTX 50 mg/m^2^ IM)** **(5000 IU/L ≤ β-hCG < 9999 IU/L)**	MTX is given on days 1 and 4 measurement of β-hCG on days 1 and 7 and if the difference between days 1 and 7 is below 15%, the 3 ${}^{\text{rd}}$ dose is given
**Multiple dose** **(1 mg/kg IM)** ** (10,000 IU/L ≤ β-hCG)**	MTX is given on days 1, 3, 5, and 7 folinic acid (1 mg/kg IM) is also administered on days 2, 4, 6, 8 MTX is given until the β-hCG level decreases more than 15% in 48 hr, or 4 doses of MTX are shown, then β-hCG is measured weekly until undetectable
MTX: Methotrexate; β-hCG: Beta- human chorionic gonadotropin	

### Ethical considerations

This study was ethically approved by the Ethical Committee of Tehran University of Medical Sciences, Tehran, Iran (Code: IR.TUMS.IKHC.REC.1398.295). All participants signed informed consent forms to share data for scientific purposes.

### Statistical analysis

We utilized SPSS software (v. 26, IBM Corp.) for statistical analysis. Pearson's Chi-square test was used to determine the treatment outcomes' differences. After using logistic regression models, we also calculated the odds ratio (OR) with a 95% confidence interval (CI) to analyze the possible association between risk factors and EP. Furthermore, we utilized *t* tests and Analysis of variance (ANOVA) to determine the significance of mean and variance differences. P < 0.05 were deemed statistically significant. To evaluate the risk factors, we utilized cross-tabulation, Chi-square tests, and logistic regression models that were adjusted and unadjusted for age, parity, gravidity, smoking, previous EP, pelvic inflammatory disease, and pelvic surgeries, including cesarean section.

## 3. Results

Among 234 patients who had a differential diagnosis of EP; 191 cases met the eligibility criteria and were included in the study. Furthermore, 190 pregnant women and 180 nonpregnant women were included in control groups. The patients in all groups were matched for age and demographic variables (Table II).

**Figure 1:**
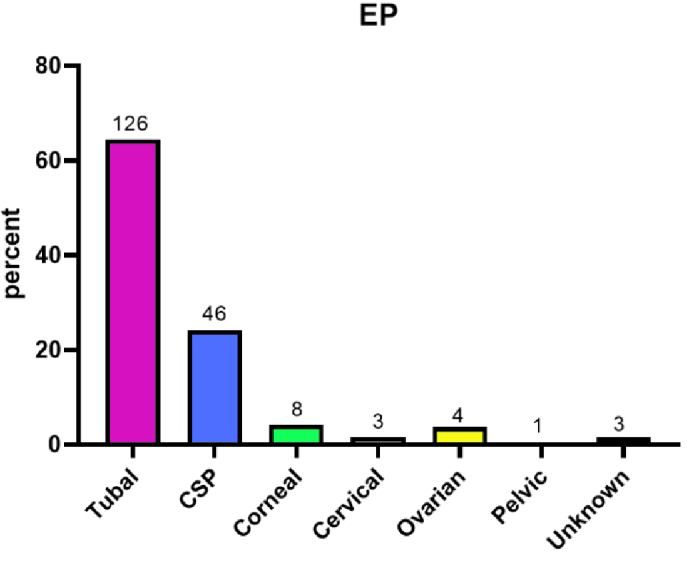
Number and percentage of subjects concerning EP site and cesarean scar pregnancy, EP: Ectopic pregnancy.

Among 191 cases, 126 patients had tubal EP, 46 had CSP and 8 had corneal EP (Figure 1). The average levels of β-hCG for expectant, medical, and surgical management were 1961, 7550, and 18,288 IU/ml, respectively. A total of 89 patients received MTX which resulted in 12 failures and 25 patients were only observed (Table III).

Results show that the average β-hCG levels prior to treatment, gestational age and parity were higher among corneal and cesarian scar in case group (Table IV).

Overall, 191 diagnosed EP cases were selected for the study. Based on our inclusion and exclusion criteria, we also selected 180 subjects for nonpregnant and 190 for intrauterine pregnancies after evaluation.

On average, our cases were 31.7 yr old; their demographic and gestational information is provided in table III. Out of the 191 cases, 90 (including failed cases) underwent surgery, 89 received MTX, and 25 underwent observation only. The demographics and characteristics are compared with various sites. Table II demonstrates that certain characteristics make subjects more susceptible to certain ectopic pregnancies. In addition, the levels of β-hCG, gestational age, and parity in corneal and scar pregnancies are significantly higher (Figure 1).

One of the primary objectives of this study was to determine the relationship between the proposed risk factors and EP. To this end, we compared the cases to 2 randomly selected control groups (intrauterine and nonpregnant). Only tubal EPs and scar pregnancies were analyzed because only these 2 categories had sufficient sample sizes for statistical analysis (Table V).

Regarding medical treatment failure, out of the 89 medically treated cases, we observed 12 failures. The results show that the cesarean section scar site yielded the highest failure rate, with 46.7%, followed by the cornea (16.7%) and tubal EP (6.3%) (27).

In terms of the type of surgery, 41 cases of tubal pregnancy were treated with salpingectomy, while only 7 cases were treated with salpingostomy. We also evaluated the cases of CSPs who received potassium chloride injections; only one of 5 CSPs who received KCl injections failed and required a second injection. The failure of medical treatment may necessitate invasive surgery by the gynecologist. In our study, 12 cases did not respond to medical treatment, 4 underwent suction and curettage (cesarean scar pregnancy), 7 underwent salpingectomy, and 1 underwent fetal reduction (corneal pregnancy) (Table V).

To evaluate the efficacy of our clinical protocol and the role of additional doses, we assessed the protocol using failure rates for various EP types. The overall analysis revealed that the additional dose could increase the success rate of both scar and tubal EPs. Since this data was a paired dependent sample and we only had sufficient samples in the tubal group to conduct a reliable test, we conducted McNemar's test in a 2 × 2 contingency table in the tubal group to determine whether the hypothesized situation in which surgery was indicated for cases who received an extra dose could be avoided. The significance of this evaluation confirmed the null hypothesis. This indicates that a higher dosage could reduce the likelihood of medical treatment failure and the need for invasive procedures. We continued our examination using the student's *t* test to determine if an additional dose was required. The results revealed that the primary difference between the 2 groups was in gestational age, which was almost 1.5 wk older in the group that required the additional dose (Tables VI and VII).

7 cases initially suspected of ovarian EP based on ultrasonographic evaluation were included in our study; one was successfully treated with MTX, 3 turned out to be tubal upon laparoscopic evaluation, 2 underwent salpingectomy, and one underwent salpingostomy. 3 cases had a gestational sac in their ovaries, resulting in the reduction of the fetus. Histopathological examination subsequently confirmed the diagnosis. Notably, 3 cases had a positive history of ART, but the number of cases was insufficient to conduct a reliable statistical analysis.

The corneal pregnancy cases included 8 subjects. 6 cases were administered MTX, one of which resulted in a fetal reduction, and one failed case underwent hysteroscopic contraceptive removal. 2 additional cases underwent laparoscopic surgery. Regarding risk factors, only one case had an EP and salpingectomy history.

3 cases of cervical pregnancy were managed during the course. One was only observed, while the other was treated medically and surgically.

**Table 2:** Participant's baseline characteristics


**Variables**	**Case group (n: 191)**	**Control (nonpregnant) (n: 180)**	**Control (pregnant) (n: 190)**	**P-value**
Age (yr)*	31.7 ± 5.428	32.97 ± 4.273	32.55 ± 6.382	0.50
Average gravidity*	2.74 ± 1.153	2.45 ± 1.276	2.26 ± 1.325	< 0.34
Average parity*	1.14 ± 1.256	1.14 ± 1.012	1.52 ± 1.725	< 0.30
History of smoking*	24 (12.6)	6 (3.3)	5 (2.6)	< 0.001
History of laparotomy**	11 (5.8)	14 (7.7)	8 (4.2)	0.35
History of TL**	10 (5.2)	7 (3.9)	1 (0.5)	0.02
Previous EP**	29 (15.2)	2 (1.1)	5 (2.6)	< 0.001
Previous salpingectomy**	45 (23.6)	0 (0.0)	0 (0.0)	< 0.001
History of OCPs**	24 (12.6)	13 (7.2)	6 (3.2)	< 0.001
History of LNG**	35 (18.3)	8 (4.4)	5 (2.6)	< 0.001
History of IUD**	14 (7.3)	4 (2.2)	2 (1.1)	< 0.001
History of ART**	25 (13.1)	15 (8.3)	12 (6.4)	0.06
*Data presented as Mean ± SD. *t *test, **Data presented as n (%). Chi-square test. TL: Tubal ligation, EP: Ectopic pregnancy, OCP: Oral contraceptives, LNG: Levonorgestrel, IUD: Intrauterine device, ART: Assisted reproductive technologies, β-hCG: Beta-human chorionic gonadotropin				

**Table 3:** The frequency of participant's based on EP site


**EP site** **Treatment**	**Surgery**	**Medical**	**Failure**
**Tubal**	46 (37.4)	60 (48.8)	2 (4.8)
**Scar **	34 (73.9)	8 (17.4)	7 (63.6)
**Cornea**	3 (37.5)	5 (62.5)	0 (0.0)
**Ovary**	3 (42.9)	2 (28.6)	0 (0.0)
**Cervix**	1 (33.3)	1 (33.3)	0 (0.0)
Data presented as n (%). EP: Ectopic pregnancy			

**Table 4:** Comparison of demographic information in case group based on EP site


**Variables**	**Tubal**	**Scar**	**Cornea**	**Ovary**	**Cervix**	**P-value**
Age (yr)*	31.25 ± 6.395	33.28 ± 5.282	34.75 ± 4.528	26.57 ± 6.399	33.33 ± 5.774	0.070
Gravidity*	2.62 ± 1.479	3.37 ± 1.271	3.38 ± 1.408	1.14 ± 0.378	2.67 ± 0.577	0.001
Parity*	1.07 ± 1.030	1.61 ± 0.714	0.88 ± 0.641	0.14 ± 0.378	1.67 ± 0.577	< 0.001
β-hCG (mIU/mL)*	5931.20 ± 12500.09	25144.98 ± 50389.94	40650.43 ± 43150.90	3968.00 ± 6348.153	5855.33 ± 8731.76	0.002
GA* (wk)	6.53 ± 1.69	6.95 ± 1.64	7.67 ± 1.86	5.71 ± 1.11	7.33 ± 2.30	0.035
Smoking**	16 (13.0)	5 (10.9)	2 (25.0)	0 (0.0)	1 (33.3)	0.77
Laparotomy**	8 (6.5)	2 (4.3)	0	0	0	0.273
TL**	7 (4.3)	2 (4.3)	1 (12.5)	0	0	0.972
Previous EP**	21 (17.1)	7 (15.2)	1 (12.5)	0	0	0.895
**Previous** salpingectomy**	18 (14.6)	3 (6.5)	1 (12.5)	0	0	0.765
OCPs**	13 (10.6)	8 (17.4)	2 (25.0)	0	1 (33.33)	0.623
LNG**	22 (17.9)	8 (17.4)	4 (50.0)	0	1 (33.33)	0.305
IUD**	8 (7.3)	5 (10.9)	0	0	0	0.604
ART**	17 (13.8)	2 (4.3)	1 (12.5)	3 (75.0)	0	0.011
*Data presented as Means ± SD. One way ANOVA test, **Data presented as n (%). Chi-square test, GA: Gestational age, TL: Tubal ligation, EP: Ectopic pregnancy, OCP: Oral contraceptives, LNG: Levonorgestrel, IUD: Intrauterine device, ART: Assisted reproductive technologies, β-hCG: Beta-human chorionic gonadotropin						

**Table 5:** Results of quantitative analysis and logistic regression models concerning different EP categories


**Tubal**							
**Risk factors**	**Nonpregnant (n = 180)**			**IUP (n = 190)**	**Case (tubal) (n = 126)**		**P-value**
**Smoking**	6 (3.3)			5 (2.6)	16 (13.0)		< 0.001
**Tubal ligation**	7 (3.9)			1 (0.5)	6 (4.3)		0.024
**Previous EP**	2 (1.1)			5 (2.6)	22 (17.1)		< 0.001
**Previous salpingectomy**	2 (1.1)			3 (1.6)	18 (14.6)		< 0.001
**OCPs (low-dose estrogen)**	13 (7.2)			6 (3.2)	13 (10.6)		0.030
**LNG**	8 (4.4)			5 (2.6)	23 (17.9)		< 0.001
**IUD**	4 (2.2)			3 (1.6)	9 (7.3)		0.005
**PID**	1 (0.6)			1 (0.5)	4 (3.1)		0.048
**ART**	9 (5.0)			7 (3.7)	18 (13.8)		0.003
**Univariate logistic regression models**							
	**Nonpregnant**				**IUP**		
**Risk factors**	**OR**		**CI**	**P-value**	**OR**	**CI**	**P-value**
	**Smoking**	4.336		0.36-8.46	0.003	5.533	1.07-9.56	0.001
**Tubal ligation**	1.491		0.01-8.04	0.466	11.405	7.89-19.45	0.024
**Previous EP**	18.324		11.4-45.23	0.000	7.618	7.52-25.67	< 0.001
**Previous salpingectomy**	15.257		8.11-38.66	0.000	10.686	8.35-26.37	< 0.001
**PID**	1.467		0.4-10.13	0.787	0.855	0.02-3.38	0.870
**OCPs**	1.518		0.67-9.64	0.310	3.624	1.28-5.23	0.011
**LNG**	4.683		2.07-12.67	< 0.000	8.059	5.28-10.97	< 0.001
**IUD**	3.061		0.5-10.12	0.073	6.504	4.93-11.69	0.019
**ART**	3.047		0.7-10.46	0.010	4.193	3.47-9.20	0.02
**Scar pregnancy**							
**Risk factors**	**Nonpregnant**			**IUP**	**Case (n: 46)**	**Chi-square**	**P-value**
**Smoking**	6 (3.3)			5 (2.6)	5 (10.9)	7.022	0.03
**Tubal ligation**	7 (3.9)			1 (0.5)	4 (4.3)	5.288	0.071
**Previous EP**	2 (1.1)			5 (2.6)	7 (15.2)	22.996	< 0.001
**Previous salpingectomy**	2 (1.1)			3 (1.6)	3 (6.5)	25.906	0.052
**OCPs (low-dose estrogen)**	13 (7.2)			6 (3.2)	8 (17.4)	11.984	0.002
**LNG**	8 (4.4)			5 (2.6)	8 (17.4)	17.073	< 0.001
**IUD**	4 (2.2)			3 (1.6)	5 (10.9)	15.244	0.004
**PID**	1 (0.6)			1 (0.5)	3 (6.5)	18.263	0.001
**ART**	9 (5.0)			7 (3.7)	2 (4.3)	1.113	0.573
**Multivariate logistic regression models**							
	**Nonpregnant**				**IUP**		
**Risk factors**	**OR**	**CI**		**P-value**	**OR**	**CI**	**P-value**
	**Smoking**	3.537	0.79-8.84		0.045	4.512	1.27-10.56	0.022
**Tubal ligation**	1.123	0.01-8.36		0.887	8.591	6.89-18.65	0.082
**Previous EP**	15.974	8.46-49.32		0.001	6.641	7.22-23.94	0.002
**Previous salpingectomy**	5.209	7.98-27.34		0.061	4.349	7.75-25.34	0.078
**PID**	12.488	1.77-14.84		0.031	3.937	1.02-4.78	0.105
**OCPs**	2.704	0.4-10.13		0.040	3.624	1.02-7.23	0.011
**LNG**	4.526	2.27-13.87		0.004	8.059	3.28-11.95	< 0.001
**IUD**	5.366	0.51-12.62		0.015	6.504	3.93-12.82	0.019
**ART**	0.864	0.72-12.46		0.855	1.188	2.47-7.18	0.833
Data are expressed as numbers and percentages. EP: Ectopic pregnancy, IUP: Intrauterine pregnancy, LNG: levonorgestrel, PID: Pelvic inflammatory disease; OCPs: Oral contraceptives (low dose estrogen), IUD: Intrauterine device, ART: Assisted reproductive technologies							

**Table 6:** Assessment of medical treatment protocol


**Tubal EP**			
**Originally planned treatment**	**Success-w/o extra dose (percent)**	**Failure-w/o extra dose (percent)**	**P-value**
**Single**	49-38 (98%-76.0%)	1-12 (2.0%-24%)	
**Double**	4-3 (80%-60%)	1-2 (20%-40%)	
**Multiple**	7 (77.8 %)	2 (22.2%)	
**Total**	60 (93.8%)	4 (6.3%)	W/O: 0.559 (0.756)
With: 7.073 (0.029)			
**Cesarean scar EP**			
**Single**	4-3 (100%-75%) ${}^{*}$	0-1 (0.0%-25%)		
**Double**	1 (100%)	0 (0.0%)	
**Multiple**	3 (30%)	7 (70%)	
**Total**	8 (53.3%)	7 (46.7%)	W/O: 3.549 (0.170)
With: 6.562 (0.038)			
**Corneal pregnancies**			
**Single**	1 (100%)	0 (0.0 %)		
**Multiple**	4 (80%)	1 (20%	
**Total**	5 (83.3 %)	1 (16.7%)	0.240 (0.624)
*Only one subject received an extra dose in scar pregnancy subjects. Chi-square, EP: Ectopic pregnancy			

**Table 7:** Assessment of the efficacy of extra dose of MTX


**Tubal EP**				
**Planned treatment**	**Extra dose**	**Success**	**Failure**	**P-value**
	Negative	38 (97.4)	1 (2.6)	
**Single**	Positive	11 (100)	0 (0.0)	0.288 (0.592)
		negative	3 (75)		1 (25)	
**Double**	Positive	1 (100)	0 (0.0)	0.313 (0.576)
	**Multiple**	Negative	7 (77.8)		2 (22.2)	
	Negative	48 (92.3)	4 (7.7)	
**Total**	Positive	12 (100)	0 (0.0)	0.985 (0.321)
	**Single-dose tubal EP**				
	Non-hypothesized failure	No	Yes	Total hypothesized
	No	38 (77.6 )	0 (0.0)	38 (76.0 )
	Yes	11 (22.4)	1 (100.0)	12 (24.0)
**Hypothesized failure**	Total non-hypothesized	49 (98.0 )	1 (2.0)	50
	**McNemar's test**	Exact significance: 0.001			
	**Characteristics**	Extra dose	Single dose	P-value
	**Gestational age**	7.64 ± 1.859	5.97 ± 1.305	0.002
**β-hCG**	1439.64 ± 1466.847	1416.74 ± 1251.83	0.959
**Mass size**	22.00 ± 9.143	28.21 ± 14.990	0.200
**Smoking (Positive history is** **counted as**	0.18 ± 0.405	0.13 ± 0.339	0.659
EP: Ectopic pregnancy, β-hCG: Beta-human chorionic gonadotropin, Chi-square				

## 4. Discussion

This study aimed to assess risk factors and treatment outcomes for surgical and therapeutic approaches to EP treatment. In addition to assessing the correlation between proposed risk factors and EP, our results revealed that emergency contraceptives, oral contraceptives, and other risk factors increase the likelihood of ectopic pregnancies in the event of a failed pregnancy.

One of the primary goals of this study was to evaluate the efficacy of the medical treatment protocol and the significance of extra doses.

The findings indicate that this method may be an option for individuals who plan future pregnancies or wish to preserve their fallopian tubes and fertility. The results showed a significant improvement in the performance of single-dose treatment plans in tubal EPs. Since gestational age was shown to influence the need for the additional dose, this modification is considered for individuals over 7.5 wk into their pregnancies (11). In a 2017 study, a meta-analysis indicated that the success rate difference between single doses and multiple doses was not statistically significant. However, side effects were more prevalent in the multiple-dose case. Consequently, their analysis indicates that the double regimen is an effective and secure alternative to the multiple-dose and single-dose protocols (1, 28-30).

A 2003 meta-analysis concluded that no statistically significant differences exist between the outcomes of multiple-dose and single-dose protocols. Nonetheless, the multiple-dose regimen is more likely to cause adverse effects (29). Regarding β-hCG concentrations and failure rate, their outcomes are also consistent with the present study. Likewise, they concluded that higher β-hCG levels increase the failure rate (23). The subtle factor that may have influenced the results is that clinicians are not blind to the factors that may result in a poor prognosis for medical treatment; consequently, they tend to select multiple-dose protocols for cases with higher β-hCG levels and a relatively poor prognosis. Based on the β-hCG concentration, the clinicians in our facility determined the medical treatment protocol. A multiple-dose protocol was designed for cases with more than 10,000 IUs who did not wish to undergo surgical management, whereas double and single doses were chosen for cases with levels below 10,000 IUs and 5000 IUs, respectively. A prospective randomized study conducted in Turkey confirms the same findings and issues attributable to inconsistent results (4, 19, 24, 30, 31). In cases where the decline in β-hCG is neither satisfactory nor significantly low, our findings and previous research suggest that an additional dose may be necessary (24, 32-35).

Regarding our findings regarding risk factors, specifically pharmaceutical contraceptives, we hypothesize that using contraceptives reduces the likelihood of both IUP and EP. However, it significantly increases the chances of EP if it fails. This is crucial for emergency contraceptives because the correlation has not been extensively studied (12, 28, 36).

Other risk factors, including smoking, intrauterine device use, and previous surgery, were investigated, and a correlation was established (5). Furthermore, our research indicates that ovulation induction increases the likelihood of EP. In 2014, a study concluded that OCPs and LNG-EC increased the risk of EP by up to 4 times that of women who did not use contraception and by up to 7 times that of women whose contraception failed (10, 22).

Several additional cases of ectopic pregnancies with LNG-EC and other emergency contraceptives have been reported (3, 5, 16). This phenomenon indicates that individuals with a positive history of contraceptive use and other risk factors and symptoms should strongly suspect EP. As an alternative medical approach, this method not only reduces the rate of surgical procedures but also decreases the possibility of infertility.

## 5. Conclusion

In summary, we conclude that the recommended clinical approach can perform better if a higher dose is administered. Our data also indicates a strong correlation between the use of contraceptives, particularly emergency contraceptives, and EP.

##  Conflict of Interest

The authors have no conflict of interest to declare.
